# Seasonal differences in intestinal flora are related to rats’ intestinal water metabolism

**DOI:** 10.3389/fmicb.2023.1109696

**Published:** 2023-02-24

**Authors:** Jing Li, Yike Sun, Ruochong Wang, Shuran Ma, Lei Shi, Kai Wang, Hairong Zhang, Tong Wang, Leilei Liu

**Affiliations:** ^1^School of Traditional Chinese Medicine, Beijing University of Chinese Medicine, Beijing, China; ^2^Department of Gastroenterology, Dongfang Hospital, Beijing University of Chinese Medicine, Beijing, China; ^3^Department of Emergency, First Teaching Hospital of Tianjin University of Traditional Chinese Medicine, Tianjin, China

**Keywords:** intestinal flora, seasonal differences, intestinal water metabolism, AQP3, AQP4, AQP8, 5-HT, VIP

## Abstract

Many studies have reported obvious seasonal differences in the intestinal flora of rats, and this stable distribution of the seasonal flora helps in maintaining the normal physiological function of the host. However, the mechanism underlying these seasonal differences in intestinal flora remains unclear. To explore the correlation among seasonal factors and intestinal water metabolism and intestinal flora, 20 Sprague Dawley (SD) rats were divided into spring, summer, autumn, and winter groups. The environment for the four seasons was simulated using the Balanced Temperature and Humidity Control system. The intestinal water metabolism was evaluated by determining the intestinal transmission function, fecal water content, water content of colonic tissue, and the colonic expression levels of AQP3, AQP4, and AQP8. The composition and relative abundance of intestinal microflora in rats in each season were assessed through 16S rDNA amplifier sequencing, and the relationship between the dominant flora and intestinal water metabolism in each season was analyzed using Spearman correlation analysis. The high temperature and humidity season could lead to an increase in intestinal water metabolism and intestinal water content in rats, whereas the low temperature and humidity season could lead to a decrease, which was closely related to the change in microflora. To explore the molecular mechanism of seasonal changes in intestinal water metabolism, the concentration of colonic 5-HT, VIP, cAMP, and PKA associated with intestinal water metabolism in rats were also examined. Seasonal changes could affect the concentration of colonic 5-HT and VIP in rats, and then regulate AQPs through cAMP/PKA pathway to affect the intestinal water metabolism. These results suggest that seasonal factors affect the level of intestinal water metabolism in rats and result in seasonal differences in intestinal flora.

## Introduction

The gut has hundreds of millions of flora, and they are closely linked to the host’s metabolism. Research on the relationship between intestinal flora and body health and disease has recently received considerable attention by the scientific community, and the composition of intestinal flora is closely related to the host environment has been confirmed (Ver Heul et al., 2019; Rutsch et al., 2020). Most current studies have focused on the effects of the gut flora on the host’s physiological function and the role they play in disease formation and the development. For example, physiologically, intestinal flora metabolites can act on the body’s emotional center through the intestine–cerebral axis, thereby affecting the host’s emotional activity (Jenkins et al., 2016); the intestinal flora can also affect the function of intestinal macrophages through n-butyrate secretion and participate in intestinal immunity (Chang et al., 2014). Pathologically, intestinal flora disorder is closely related to the formation and development of various intestinal diseases, such as irritable bowel syndrome (IBS; Wang et al., 2020), inflammatory bowel disease (Du et al., 2022), and colorectal cancer (Zou et al., 2018). Abnormal intestinal flora can also cause many parenteral diseases, such as asthma (Salameh et al., 2020), diabetes (Du et al., 2022), and nonalcoholic fatty liver (Di Ciaula et al., 2022). Therefore, researchers are keenly interested in understanding the reasons for the change in the intestinal flora composition.

The alimentary canal is directly connected to the external world, and external environmental factors, such as circadian rhythm (Liang et al., 2015), seasonal rhythm (Liu et al., 2018), air pollution (Ran et al., 2021), and diet (De Angelis et al., 2019), can easily affect the intestinal flora, thereby leading to changes in the host’s physiological functions. Of these factors, the effect of seasonal changes on intestinal flora is among the current research hotspots (Segawa et al., 2005; Zhu et al., 2020), through changes in composition and diversity, microbiota can adapt to seasonal changes in the environment and maintain host health (Davenport et al., 2014; You et al., 2022). However, how seasonal factors act on the flora and lead to changes in its composition remains unclear. After literature review, we found that the aqueous environment, as among the necessary conditions for the survival of flora, is closely related to the changes in the composition of flora (Or et al., 2007; Dechesne et al., 2008; Young et al., 2008; Dechesne et al., 2010; Vos et al., 2013). On the other hand, both the aqueous environment and the flora composition vary seasonally (Lv et al., 2021). Therefore, the present study mainly attempts to understand the relationship between host’s intestinal water metabolism and seasonal differences in intestinal flora.

Temperature and humidity are common external environmental factors affecting body’s water metabolism. For example, high temperatures (30°C ± 1°C) increase moisture loss caused by respiration of insects and epidermal transpiration (Kleynhans et al., 2014), and high humidity (90% ± 2%) can increase blood urea nitrogen and antidiuretic hormone secretion, leading to water metabolism disorders (Yin et al., 2022). Clinical studies have also reported that diarrheal diseases are frequently due to relatively high temperatures and humidity in summer and autumn (Phung et al., 2015; Anwar et al., 2019), and these diseases are often accompanied by intestinal flora disorders (Li et al., 2021). Thus, environmental factors, especially temperature and humidity, are reasonably speculated to affect intestinal water metabolism, thereby causing changes in the intestinal flora composition. Based on the environmental characteristics of the four seasons in Beijing, this study strictly controlled the environmental variables and mainly explored the intrinsic connection between seasonal changes in rats’ intestinal flora and intestinal water metabolism ability from the perspective of temperature and humidity.

## Materials and methods

### Animal and experimental grouping

Twenty male specific-pathogen free (SPF)-grade Sprague–Dawley (SD) rats (age: 6 weeks; weight: 200 ± 20 g) were purchased from SPF (Beijing) Biotechnology Co., Ltd. Number of animal license: SYXK (Beijing) 2019–0010. This study was approved by the Ethics Committee of Beijing University of Chinese Medicine (approval number: BUCM-4-2,021,032,603-1,059).

All rats were adaptability housed for 7 days and randomly divided into four groups (n = 5 rats each) according to the method of the random number table. All rats were fed sterilized feed and deionized water in an artificial climate simulator (NHRHG6, Chongqing Hongrui Experimental Instrument Co., Ltd).

The artificial climate simulator uses a Balanced Temperature and Humidity Control (BTHC) system to control temperature (T) and relative humidity (RH). One can also design the corresponding parameters according to the actual environmental and climatic characteristics of the area and simulate the local climate. By strictly controlling environmental variable factors, the interference of other factors in experimental results can be reduced. Referring to the data^1^ displayed by the Spanish National Meteorological Institute (Agencia Estatal de Meteorología, AEMET), select the China-Beijing area, we established the environment temperature for the four seasons based on the information about the average monthly maximum temperature for 2020. The temperatures of the four seasons were 14.8°C in spring, 26.2°C in summer, 12.9°C in autumn, and − 2.3°C in winter. Then, the relative humidity was established with reference to the average humidity of the natural season in 2020. Accordingly, the humidity of the four seasons was 40.3% in spring, 64.3% in summer, 52.2% in autumn, and 41.2% in winter.

### 16S rDNA amplicon sequencing of intestinal flora

The experiment ended after 4 groups of rats were fed in the artificial climate simulator for 1 month. After the experiment ended, 3 rats were randomly selected from each group and their perianal skin was disinfected with 75% alcohol. The feces of each rat was collected using sterilized forceps and placed into a sterile 2-mL Eppendorf tube. The collected feces were immediately stored in an ultra-low temperature refrigerator at −80°C.

The microbial community genomic total DNA was extracted by E.Z.N.A. ® Stool DNA Kit (Omega Bio-Tek, United States). PCR amplification was performed using 16S V4 region primers (515F and 806R) according to the requirement of Phusion® High-Fidelity PCR Master Mix amplification kit (New England Biolabs). The purity of the PCR amplified sample was tested by agarose gel electrophoresis. After passing the test, the instructions of the TruSeq® DNA PCR-Free Sample Preparation Kit (New England Biolabs) were followed to build a sequencing library. After building and testing the library, the NovaSeq6000 sequencing platform was used by Beijing Novogene Technology Co., Ltd., to perform the sequencing of the flora. After sorting out the original data, effective tags were clustered, and the sequences were clustered into operational taxonomic units (OTUs) with 97% consistency. Then, using the representative sequences of OTUs, species annotation (Venn Graph, species relative abundance column accumulation map, species abundance cluster heat map), alpha diversity analysis (Shannon index, Chao1 index), beta diversity analysis (principal co-ordinate analysis, PCoA), and between-group difference species analysis (LDA effect size analysis) were performed.

### Fecal water content test

After the experiment, each rat was placed in a metabolic cage, and their feces were collected for 24 h. The feces morphology was assessed according to the Bristol Stool Form Scale (BSFS; Table 1).The feces weight was recorded as the fecal wet weight. Then, all the fecal samples were dried with a filter paper, placed in an electric blast drying oven (GZX-9023MBE, Shanghai Boxun Industrial Co., Ltd), and dried at 60°C. Then, the fecal dry weight was recorded. The fecal water content was calculated as follows: the fecal water content = (fecal wet weight – fecal dry weight)/fecal wet weight × 100%.

**Table 1:** Bristol Stool Form Scale.

Type	Form
I	Separate hard lumps, like nuts
II	Sausage-shaped but lumpy
III	Like a sausage or snake but with cracks on its surface
IV	Like a sausage or snake, smooth and soft
V	Soft blobs with clear-cut edges
VI	Fluffy pieces with ragged edges, a mushy stool
VII	Watery, no solid pieces

### Detection of intestinal transmission function

Distilled water (800 ml) was added to 100 g gum arabic (A108975-500G, Beijing Meikang Instrument Equipment Co., Ltd). Boiled the solution until transparent. Then, 50 g of activated carbon (C139601, Beijing Meikang Instrument Equipment Co., Ltd) was added to the solution and boiled 3 times. After the solution was cooled, distilled water was subsequently added to make the final volume of the solution to 1,000 ml. After each rat was placed in a metabolic cage, they received a gavage of 2 ml of the 100 g/l activated carbon suspension. The time from the completion of the activated carbon gavage to the excretion of the rat’s first black stool was recorded as the fecal excretion time.

### Water content of colonic tissue test

All the rats were sacrificed through cervical dislocation. Then, 2 cm of the colonic tissue above the anus of each rat was cut off with sterilized surgical scissors and weighed after being dried with a filter paper. This weight was recorded as the wet weight of colonic tissue. The collected tissue was then dried in the electric blast drying oven at 60°C and weighed. This weight was recorded as the dry weight, and the water content of the colonic tissue was calculated as follows: water content of colonic tissue = (wet weight of colonic tissue − dry weight of colonic tissue)/wet weight of colonic tissue × 100%.

### Colonic AQP, 5-HT, VIP, cAMP, and PKA expression level detection

The colonic tissue (2 cm) of the rats was frozen in liquid nitrogen and stored at −80°C for later use. The tissue was then made into homogenates, and the supernatant was used in accordance with the instructions of Aquaporin 3 (MB-2017A, Jiangsu Enzyme Biotechnology Co., Ltd), Aquaporin 4 (MB-2016A, Jiangsu Enzyme Biotechnology Co., Ltd), Aquaporin 8 (MB-7036A, Jiangsu Enzyme Biotechnology Co., Ltd), 5-Hydroxytryptamine (UK Abcam), Vasoactive intestinal polypeptide (American Raybio), Cathelicidin Antimicrobial Peptide (Shanghai Bluegene Biotechnology Co., Ltd) and Protein kinase A (Shanghai Bluegene Biotechnology Co., Ltd) ELISA kits. Later, the absorbance (optical density (OD) value) of each well was measured sequentially at 450 nm. The standard curves were drawn according to the standard concentration and OD value given in the instructions, the formula was established, and the concentration of each sample was calculated using the formula.

### Correlation analysis

The correlation between the relevant indicators of intestinal water metabolism (fecal excretion time, dry and wet weights of colonic tissue, water content of colonic tissue, fecal dry and wet weights, fecal water content, AQP3, AQP4, and AQP8) and the abundance of the dominant intestinal flora in each season was studied through Spearman correlation analysis, and the relevance heatmap was drawn according to the obtained results.

### Data analysis

SPSS20.0 software (IBM, United States) was used for statistically analyzing the data. All the data were expressed as mean ± SD. The Shapiro–Wilk test was first used for determining normality. When the data of each group followed a normal distribution and the variances were equal, one-way ANOVA was performed to compare multiple groups, and the two–two comparisons among the groups were performed using the LSD test at the same time. However, when the data of each group followed a normal distribution but the variance was unequal, the Welch analysis was performed to compare multiple groups, and the two–two comparisons among the groups were performed using the Dunnett T3 test at the same time. When the data of the groups did not follow a normal distribution, the Kruskal–Wallis test was performed to compare multiple groups, and the two–two comparisons among the groups were performed using the Mann–Whitney test at the same time. α = 0.05 was considered the test level, and *p* < 0.05 indicated that the difference was statistically significant, and *p* < 0.01 indicated that the difference was extremely significant.

## Results

### Effects of different seasons on intestinal water metabolism in rats

The feces of all groups were type IV in BSFS, the length of the feces was uniform and the color was brownish-black, and no mushy or loose stools.

### Effects of different seasons on fecal water content and water content of colonic tissue in rats

**Figure 1:**
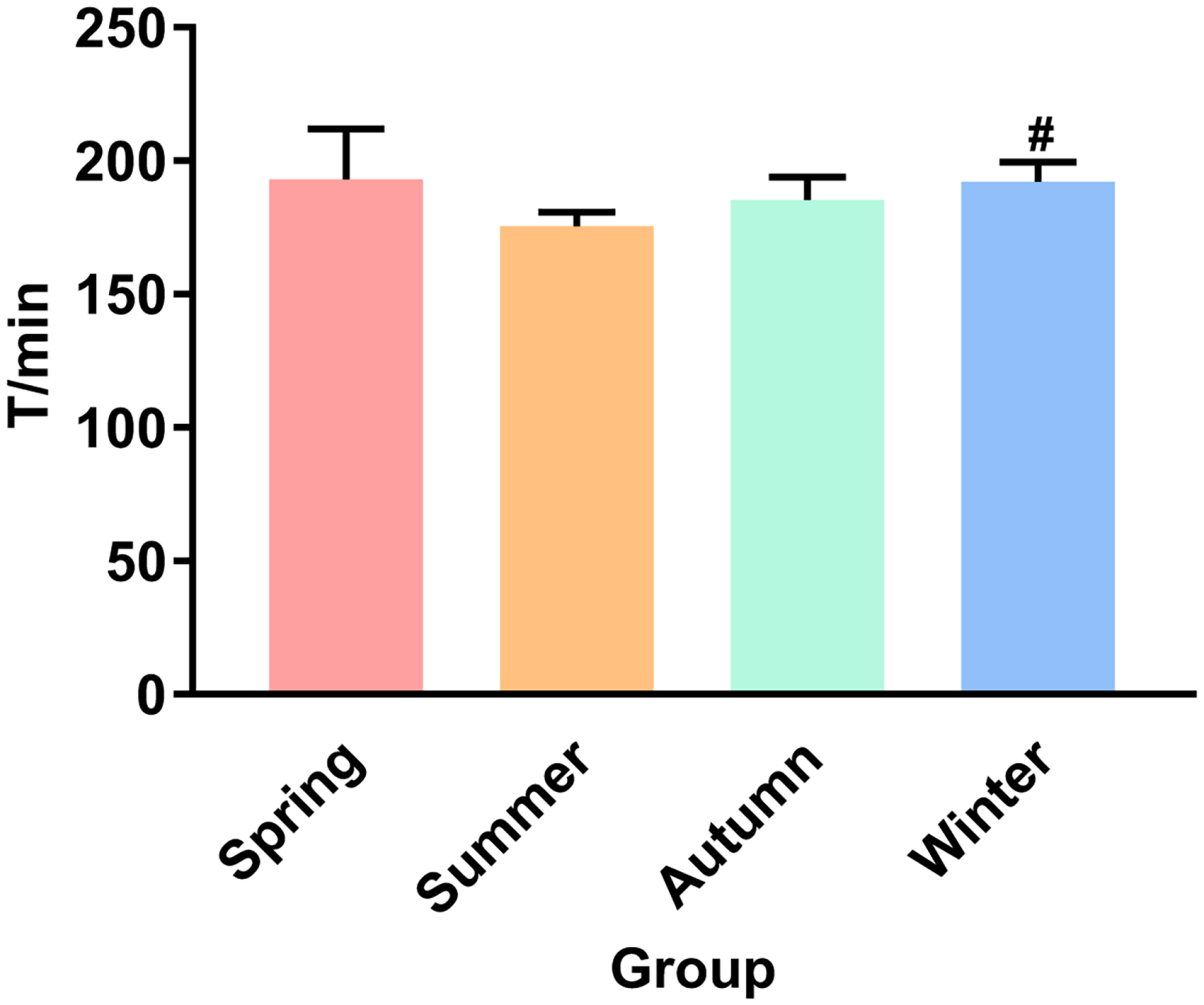
Fecal excretion time. ^#^*P* < 0.05 compared with the summer group.

Figure 1 presents the fecal excretion time. The fecal excretion time of the winter group increased significantly compared with the summer group (*p* < 0.05). Although the fecal excretion time in the summer group was not statistically different from that in the spring and autumn groups, an obvious decrease trend was observed in the summer group.

**Figure 2:**
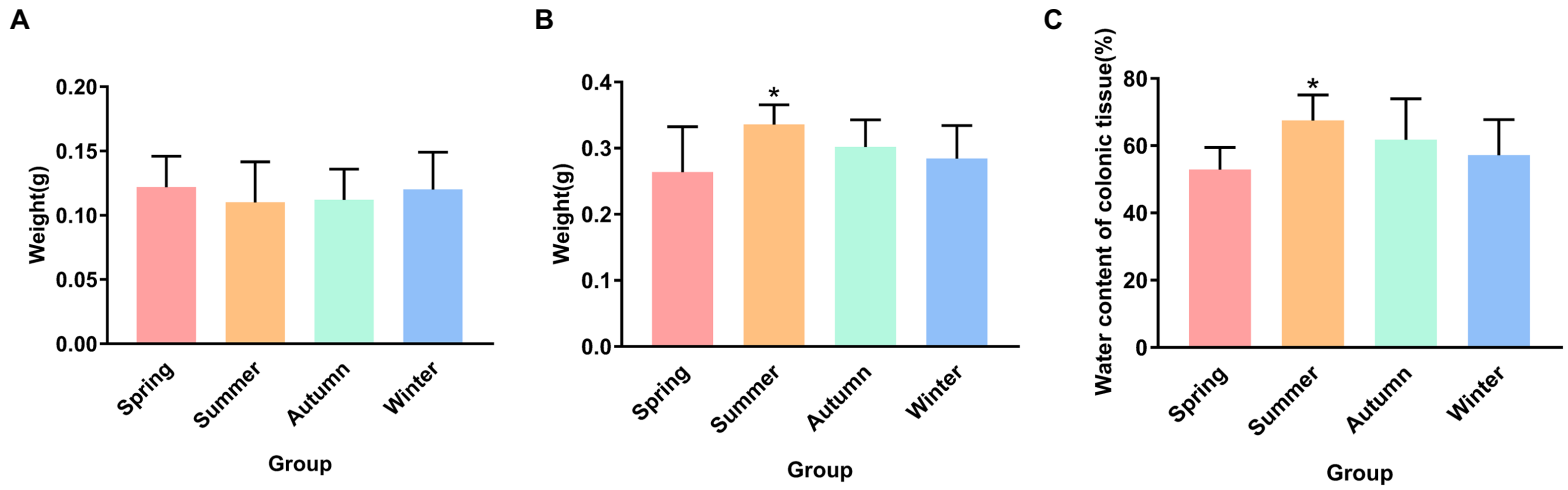
**(A)** Dry weight of colonic tissue. **(B)** Wet weight of colonic tissue. **(C)** Water content of colonic tissue. *p < 0.05 compared with the spring group.

Figure 2 presents the dry and wet weights and water content of colonic tissue in different seasons. Although no statistical difference was observed in the dry weight of colonic tissue, an increase trend was observed in the spring group compared with the other groups, and a decrease trend was observed in the summer group compared with other groups. The wet weight of colonic tissue in the summer group was the highest in four seasons, and was significantly different from that in the spring group (*p* < 0.05). Compared with the autumn group and the winter group, the wet weight of colonic tissue in the spring group showed a decrease trend. The water content of the colonic tissue in the summer group was the highest in four seasons, and was significantly different from that in the spring group (*p* < 0.05).

**Figure 3:**
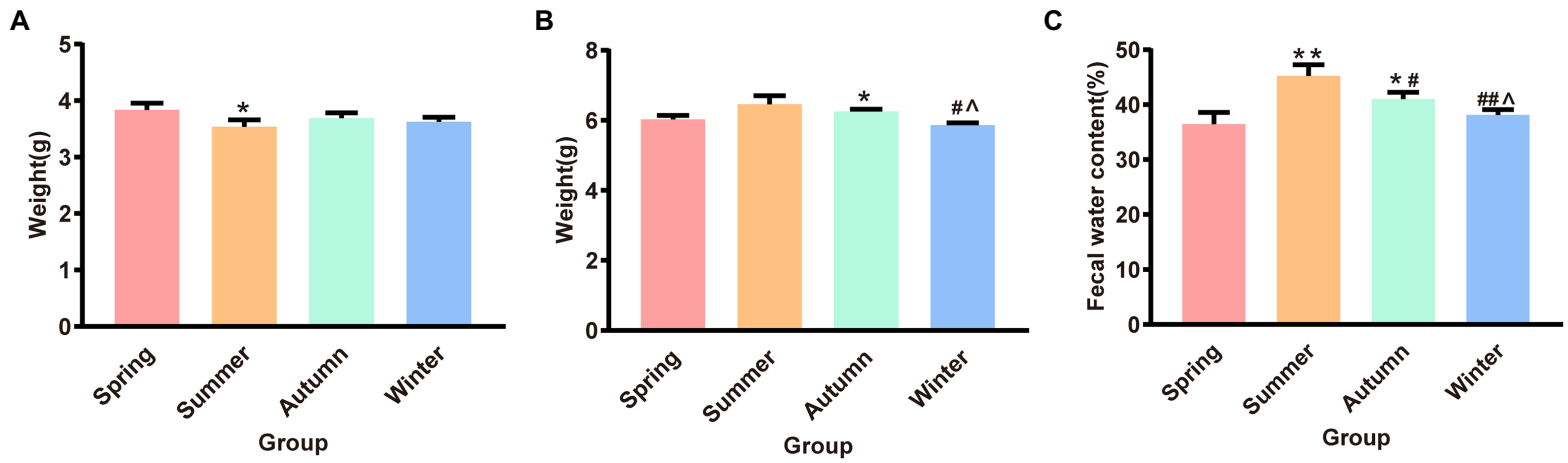
**(A)** Fecal dry weight. **(B)** Fecal wet weight. **(C)** Fecal water content. *p < 0.05 compared with the spring group; ^#^*p* < 0.05 compared with the summer group; ^##^*p* < 0.01 compared with the summer group; ^∧^*p* < 0.05 compared with the autumn group.

Figure 3 illustrates the dry and wet weights and water content of feces in different seasons. The fecal dry weight was the lowest in the summer group and was significantly different from that in the spring group (*p* < 0.05). The fecal wet weight was the highest in the summer group and the lowest in the winter group. The fecal wet weight in the winter group was significantly different from that in the summer and autumn groups (*p* < 0.05, *p* < 0.05, respectively). The fecal wet weight in the autumn group increased and was significantly different from that in the spring group (*p* < 0.05). The fecal water content in the summer group was the highest and was significantly different from that in the spring, autumn, and winter groups (*p* < 0.01, *p* < 0.05, *p* < 0.01, respectively). The fecal water content in the autumn group was the second highest and was statistically different from that in the spring and winter groups (*p* < 0.05, *p* < 0.05, respectively).

### Effects of different seasons on AQP3, AQP4, and AQP8 in rat colons

**Figure 4:**
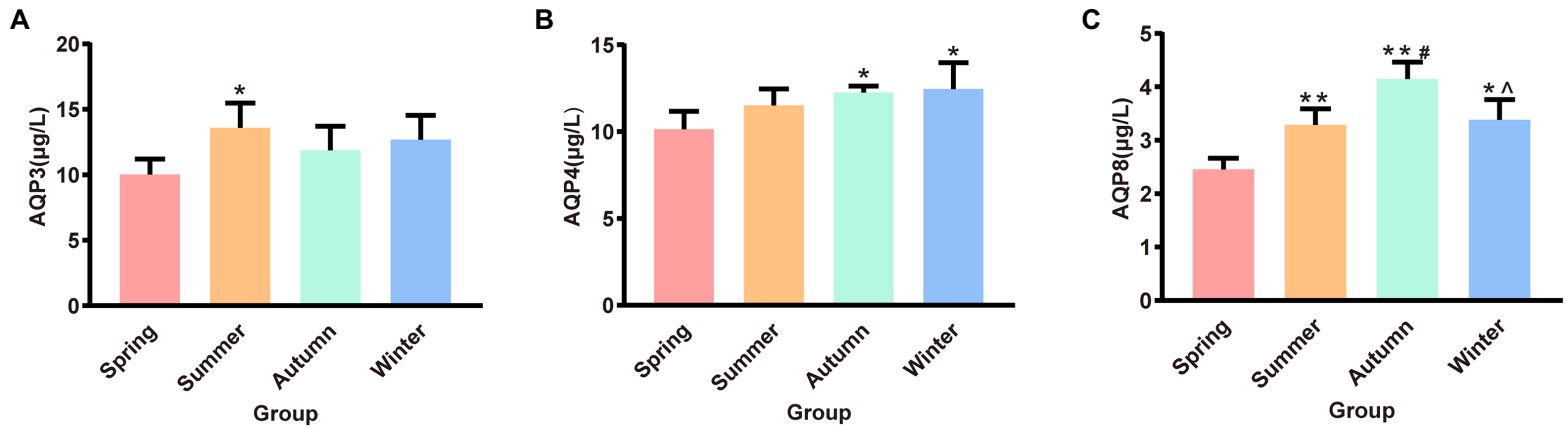
**(A)** AQP3 content of colon. **(B)** AQP4 content of colon. **(C)** AQP8 content of colon. ^*^p < 0.05 compared with the spring group; ^#^*p* < 0.05 compared with the summer group; ^**^p < 0.01 compared with the summer group; ^∧^*p* < 0.05 compared with the autumn group.

The contents of AQP3, AQP4, and AQP8 in rat colons in different seasons are presented shown in Figure 4. The content of colonic AQP3 was the highest in the summer group and the lowest in the spring group, and the difference between these two groups was statistically significant (*p* < 0.05). The content of colonic AQP4 was the lowest in the spring group and the highest in the winter group. The spring group had lower AQP4 content than the autumn and winter groups (*p* < 0.05, *p* < 0.05, respectively). The spring group had the lowest AQP8 content. The AQP8 content in the spring group was significantly different from that in the summer, autumn, and winter groups (*p* < 0.01, *p* < 0.01, *p* < 0.05, respectively). The autumn group had the highest AQP8 content, and the AQP8 content in the autumn group was higher than that in the summer and winter groups (*p* < 0.05, *p* < 0.05, respectively).

### Effects of different seasons on 5-HT, VIP, cAMP and PKA in rat colons

**Figure 5:**
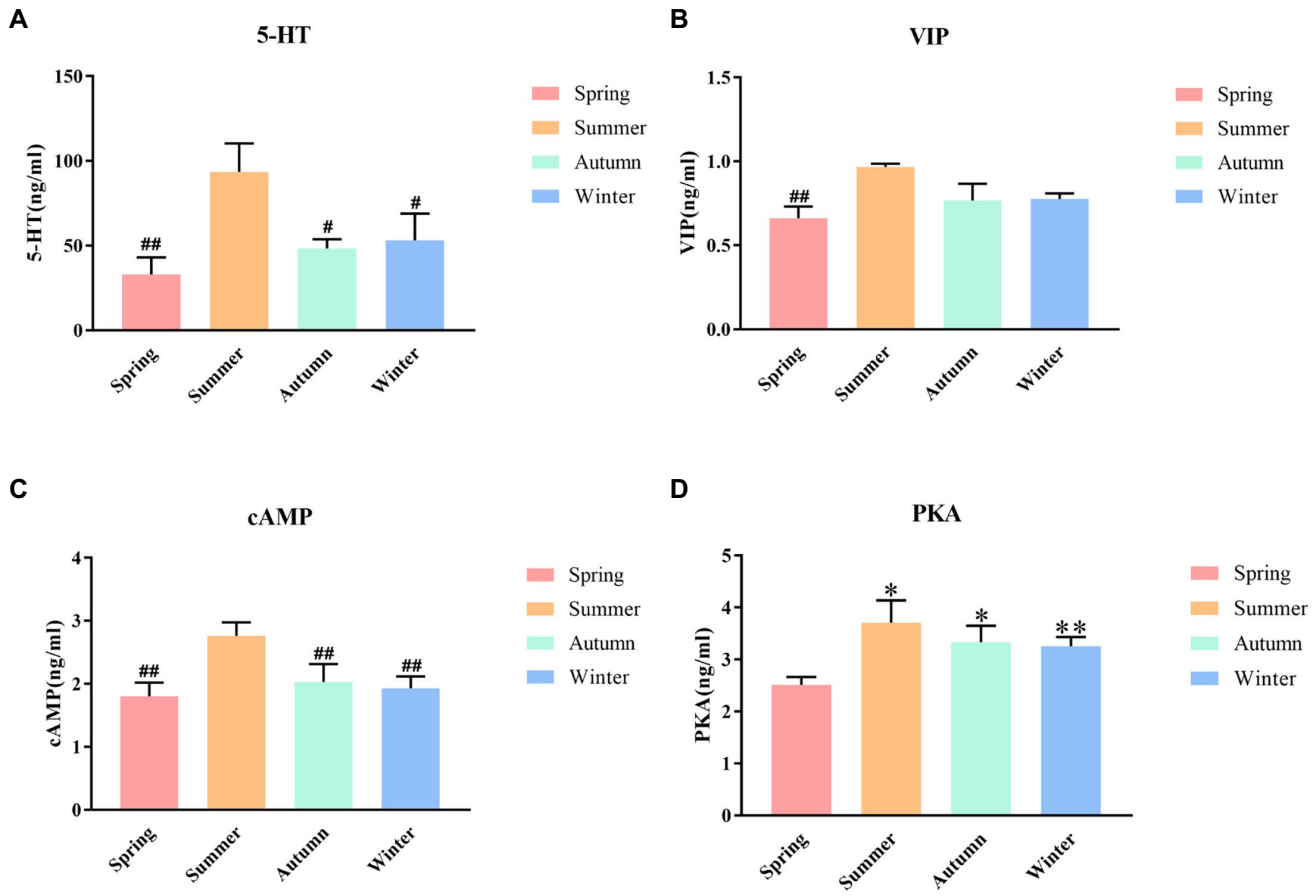
**(A)** 5-HT content of colon. **(B)** VIP content of colon. **(C)** cAMP content of colon. **(D)** PKA content of colon. *p < 0.05 compared with the spring group; **P<0.01 compared with the spring group; ^#^*P*<0.05 compared with the summer group; ^##^*P*<0.01 compared with the summer group.

The contents of 5-HT, VIP, cAMP and PKA in rat colons in different seasons are presented shown in Figure 5. The content of colonic 5-HT was the highest in the summer group. The 5-HT content in the summer group was significantly different from that in the spring, autumn, and winter groups (*p* < 0.01, *p* < 0.05, *p* < 0.05, respectively). A decrease trend was observed in the 5-HT content in the spring group compared with the autumn, and winter groups. The content of colonic VIP was the highest in the summer group and the lowest in the spring group, and the difference between these two groups was statistically significant (*p* < 0.01). The summer group had the highest cAMP content. The cAMP content in the summer group was significantly different from that in the spring, autumn, and winter groups (*p* < 0.01, *p* < 0.01, *p* < 0.01, respectively). A decrease trend was observed in the cAMP content in the spring group compared with the autumn, and winter groups. The content of colonic PKA was the highest in the summer group and the lowest in the spring group. The PKA content in the spring group was significantly different from that in the summer, autumn, and winter groups (*P* < 0.05, *P* < 0.05, *P* < 0.01, respectively).

### Effect of seasonal humidity and temperature difference on intestinal flora in rats

**Figure 6:**
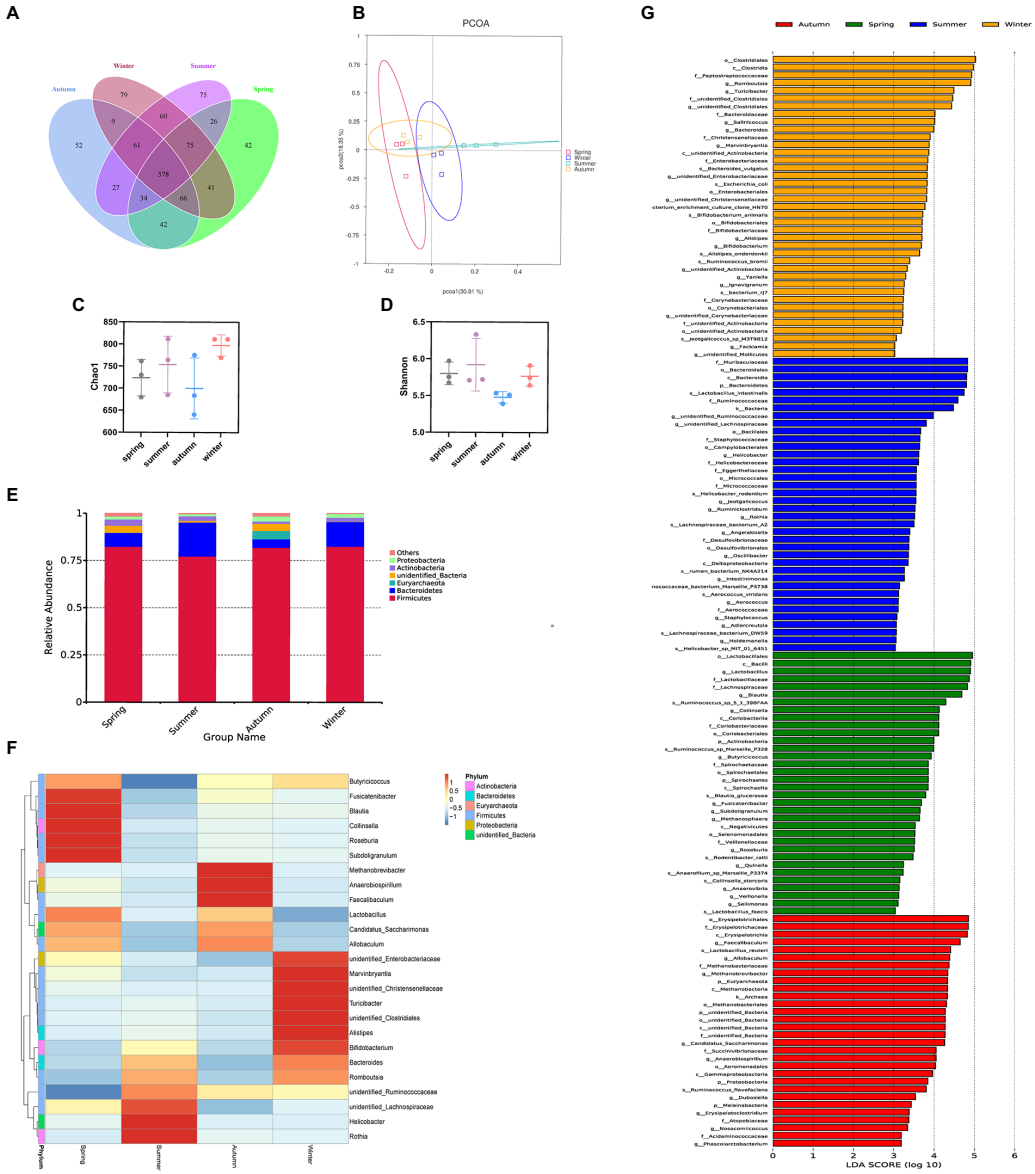
Different seasons affect the structure and composition of the intestinal flora. **(A)** The Venn diagram of spring, summer, autumn, and winter groups. **(B)** PCoA score. **(C)** Chao index. **(D)** Shannon index. **(E)** Columnar accumulation plot of relative species abundance at the phylum level: top 6 species according to the abundance were selected for each group. **(F)** Heatmap of gut microbiota at the genus level: top 25 genera according to the abundance were selected for each group (the abundance of each group was the average of all samples in the group). **(G)** The LEfSe analysis.

We obtained 665, 710, 613, and 735 OTUs in the spring, summer, autumn, and winter groups, respectively. The Venn diagram showed that all four groups shared 578 OTUs. Among them, the summer group and the winter group shared 774 OTUs, the spring group and the winter group shared 760 OTUs, the spring group and the autumn group shared 720 OTUs, the autumn group and the winter group shared 714 OTUs, the spring group and the summer group shared 713 OTUs, the summer group and the autumn group shared 700 OTUs. It was shown that in terms of intestinal flora structure, the summer group and winter group were closer (Figure 6A). The Chao1 and Shannon indices were used to calculate community richness and community diversity of the intestinal flora, respectively. The alpha diversity analysis revealed that the winter group had the highest Chao1 index, followed by the summer and spring groups, whereas the autumn group had the lowest Chao1 index (Figure 6C). The analysis also revealed that the summer group had the highest Shannon index, followed by the spring, winter, and autumn groups (Figure 6D). However, no statistically significant difference was observed in these changes. The PCoA based on OTU abundance revealed that the sample distance in each group was relatively close, which indicated that the species composition structure of each sample in each group was similar. In terms of differences between groups, the distance between the spring and autumn groups was lower than that between the other groups, indicating that the composition of intestinal flora in the spring and autumn was more similar. By contrast, the distance between the summer and the other groups was relatively greater, suggesting that the rat’s intestinal flora in summer was more distinct (Figure 6B).

Regarding the phyla, Firmicutes and Bacteroidetes were the two most dominant phyla among all samples. Firmicutes accounted for more than 75% of the flora and was the main flora. Compared with the other three seasons, the proportion of Firmicutes was relatively low in summer (77.25%; Figure 6E). Table 2 presents the detailed data of the abundance of the top 6 species in each season at the phylum level. The species abundance cluster heatmap (Figure 6F) and Table 3 show the microbes with a higher relative abundance. The microbes with a relatively high abundance in spring were *Lactobacillus and Blautia*. The microbes with a relatively high abundance in autumn were *Lactobacillus and Faecalibaculum*. The microbes with a relatively high abundance in winter and summer were *Lactobacillus and Faecalibaculum*. The subsequent LEfSe analysis revealed that the biomarkers in the spring group were *Lactobacillus*, *Blautia*, etc. The biomarkers in the summer group were *Rothia, Helicobacter*, etc. The biomarkers in the autumn group were *Faecalibaculum*, *Methanobrevibacter*, etc. The biomarkers in the winter group were *Bacteria, Romboutsia*, *Turicibacter*, and *Alistipes* (Figure 6G).

**Table 2:** The abundance of the top 6 species in each season at the phylum level.

Taxonomy	Firmicutes	Bacteroidetes	Euryarchaeota	unidentified_Bacteria	Actinobacteria	Proteobacteria
Spring	0.824316	0.072617	0.000927	0.037627	0.03282	0.01564
Summer	0.772437	0.180315	0.000097	0.00862	0.023606	0.012039
Autumn	0.818684	0.045574	0.043671	0.03847	0.011488	0.026758
Winter	0.825068	0.131255	0.00057	0.001164	0.020393	0.019835

**Table 3:** The microbes with a higher relative abundance at the genus level.

Taxon	Spring	Summer	Autumn	Winter
Lactobacillus	0.357121	0.243767	0.327314	0.197556
Romboutsia	0.058947	0.203369	0.065755	0.217385
Blautia	0.101988	0.005638	0.023569	0.022648
Faecalibaculum	0.013712	0.002789	0.096132	0.003813
Methanobrevibacter	0.000837	0.000097	0.043665	0.000091
unidentified_Clostridiales	0.0139	0.01618	0.010524	0.065609

### Correlation analysis of intestinal water metabolism and composition of intestinal flora

**Figure 7:**
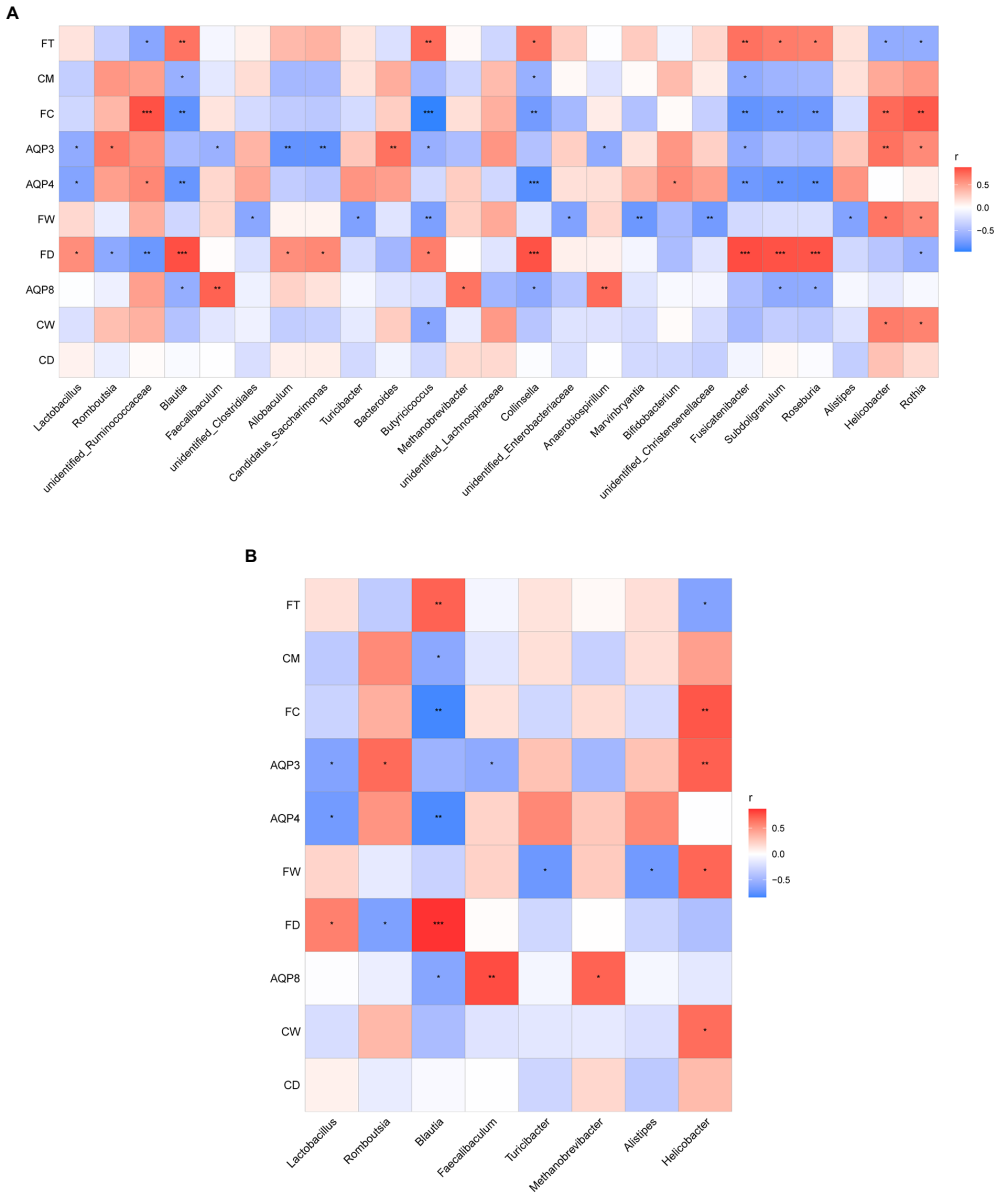
**(A)** Correlation analysis between the abundance of the top 25 microbes at the genus level and the related indexes of intestinal water metabolism. **(B)** Correlation analysis between the dominant microbes in different seasons and the related indexes of intestinal water metabolism. The ordinate is intestinal water metabolism and information on AQPs (CD, dry weight of colonic tissue; CW, wet weight of colonic tissue; CM, water content of colonic tissue; FD, fecal dry weight; FW, fecal wet weight; FC, fecal water content; FT, fecal excretion time). The abscissa is the genus level-related species information obtained through clustering. The Spearman correlation coefficient r(−1 ≤ *r* ≤ 1) is expressed in a color shade. The darker red heatmap color indicates that *r* is closer to 1. When *r* < 0, the environmental factor is considered negatively correlated with the flora, and when *r* > 0, a positive correlation is considered, *p < 0.05, **p < 0.01, ***p < 0.001.

To understand whether seasonal differences in the intestinal flora are related to intestinal water metabolism, we investigated the correlation between fecal excretion time, fecal dry and wet weights, fecal water content, dry and wet weights of colonic tissue, water content of colonic tissue, content of AQP3, AQP4, and AQP8, and dominant intestinal bacteria in the intestinal environment during the four seasons. This correlation was determined through Spearman correlation analysis. According to the results, the abundance of the top 25 flora in rat feces was correlated with changes in intestinal water metabolism (Figure 7A).

As shown in Figure 7, the dominant microbes *Lactobacillus* and *Blautia* in the spring group exhibited a certain correlation with intestinal water metabolism. The abundance of *Lactobacillus* was negatively correlated with the AQP3 content (*r* < 0, *p* < 0.05) and the AQP4 content (*r* < 0, *p* < 0.05); it was positively correlated with fecal dry weight (*r* > 0, *p* < 0.05). The abundance of *Lactobacillus* had a negative correlation trend with both fecal water content and water content of colonic tissue. The abundance of *Blautia* was significantly and negatively correlated with the AQP4 content (*r* < 0, *p* < 0.01), and the AQP8 content (*r* < 0, *p* < 0.05). The abundance of *Blautia* was positively correlated with fecal dry weight (r > 0, *p* < 0.01) and the water content of colonic tissue (*r* > 0, *p* < 0.05). The abundance of *Blautia* exhibited a significant negative correlation with fecal water content (*r* < 0, *p* < 0.01) and a significant positive correlation with fecal excretion time (*r* > 0, *p* < 0.01).

The dominant microbes *Romboutsia* and *Helicobacter* in the summer group were related to intestinal water metabolism. The abundance of *Romboutsia* was positively correlated with the AQP3 content (*r* > 0, *p* < 0.05) and negatively correlated with fecal dry weight (*r* < 0, *p* < 0.05). The abundance of *Romboutsia* also had a positive correlation trend with both fecal water content and water content of colonic tissue. The abundance of *Helicobacter* was significantly positively correlated with the AQP3 content (*r* > 0, *p* < 0.01), fecal wet weight, wet weight of colonic tissue (*r* > 0, *p* < 0.05), and fecal water content (*r* > 0, *p* < 0.01). The abundance of *Helicobacter* was negatively correlated with fecal excretion time (*r* < 0, *p* < 0.05).

The dominant microbes *Faecalibaculum* and *Methanobrevibacter* in the autumn group were related to intestinal water metabolism. The abundance of *Faecalibaculum* was positively correlated with the AQP3 (*r* < 0, *p* < 0.05) and AQP8 contents (*r* > 0, *p* < 0.01). The abundance of *Faecalibaculum* had a negative correlation trend with the water content of colonic tissue. The abundance of *Methanobrevibacter* was positively correlated with the AQP8 content (*r* > 0, *p* < 0.05), and it had a negative correlation trend with the water content of colonic tissue.

The dominant microbes *Alistipes* and *Turicibacter* in the winter group were associated with intestinal water metabolism. The abundance of *Alistipes* was negatively correlated with fecal wet weight (*r* < 0, *p* < 0.05) and fecal water content. The AQP4 content exhibited a positive correlation trend with the abundance of *Alistipes*. The abundance of *Turicibacter* was negatively correlated with fecal wet weight (*r* < 0, *p* < 0.05), and it had a positive correlation trend with AQP4 content and had a negative correlation trend with fecal water content (Figure 7B).

## Discussion

The intestinal barrier is composed of four barriers, namely the mechanical, chemical, immune, and microbial barriers, that are closely related and influence each other. The intestinal flora has a crucial role as the microbial barrier, while intestinal water metabolism is related to epithelial cells in the intestinal mechanical barrier. Colonic epithelial cells participate in water and fluid metabolism by regulating luminal secretion and fluid and ion absorption (Negussie et al., 2022). Therefore, changes in the water content of colonic tissue and fecal water content directly reflect changes in intestinal water metabolism. A study on the water balance within the Greek population revealed significant differences in water loss among different seasons under the same water balance distribution (Malisova et al., 2013). Our study showed that compared with other seasons, the water content of colonic tissue and fecal water content of rats in the summer group were higher, suggesting that intestinal water metabolism was enhanced. By contrast, the water content of colonic tissue and fecal water content of rats were lower in the spring group, which suggested that the weakening of intestinal water metabolism. This confirms that a seasonal difference exists in the level of intestinal water metabolism in rats, which may be related to the seasonal change in the intestinal water reabsorption capacity.

According to a report, intestinal water transport is related to aquaporin expression on the surface of epithelial cells (Zhang et al., 2019). AQPs is a series of highly selective transmembrane channels, in which AQP3, AQP4, and AQP8 are mainly expressed in the colonic epithelium (Mobasheri et al., 2005; He and Yang, 2019). They can regulate colonic water transport and directly affect the change in intestinal water content and the level of intestinal water metabolism. Therefore, AQP3、AQP4 and AQP8 have always been the core index to investigate the mechanism of intestinal water metabolism. The increased AQP3 expression level allows the transport of a large amount of water to the lumen side (Ikarashi et al., 2016) and increases the water content in the intestinal cavity. The increased AQP4 and AQP8 expression reduces the intestinal fluid content by reducing the fecal water content, mucus secretion, and intestinal peristalsis (Hu et al., 2019). For example, AQP3 protein expression in the colon of rotavirus-infected diarrhea mice is significantly increased, whereas AQP4 and AQP8 protein expression is downregulated (Cao et al., 2014). The decreased AQP8 expression level in the mouse colon can alleviate the symptoms of constipation (Wang L. et al., 2022). Our results also confirmed that the differential expression of AQP3, AQP4, and AQP8 in the colon is often consistent with the change in the colonic water content or fecal water content. For example, AQP3 expression in the colon of summer group rats increased, whereas AQP4 and AQP8 expression decreased, which was consistent with the increased the fecal water content and intestinal water content. However, in the spring group, the expression of AQP3, AQP4, and AQP8 decreased and the level of intestinal water metabolism decreased, which was also consistent with the decreased colonic water content in rats. Thus, the seasonal difference in water metabolism in the intestinal tract is related to the seasonal change in the AQP expression level.

Cerebroenteric peptide is an important neurotransmitter or peptide hormone transmitted between the central nervous system and the enteric nervous system. As an important factor in regulating gastrointestinal fluid balance, its role in the development of intestinal diseases has been increasingly valued. Among them, vasoactive intestinal peptide (VIP) is an effective stimulant that secretes water and electrolytes through the intestinal mucosa. Increased serum VIP can cause diarrhea (Xiong et al., 2018). 5-hydroxytryptamine (5-HT) plays an important role in regulating gastrointestinal motility and secretion. Increased secretion of 5-HT is strongly associated with the development of IBS-D (Gao et al., 2022). Studies have shown that 5-HT and VIP have a regulatory effect on AQPs, which may be achieved by cAMP/PKA signaling mechanisms (Kon et al., 2015; Tan et al., 2021). 5-HT activates adenylate cyclase (AC) by stimulating the protein Gs, which can increase cyclic adenosine monophosphate (cAMP), and then activate protein kinase A (PKA)(Galligan, 2021). The upregulated cAMP / PKA signaling contributes to phosphorylation and expression of AQP3, AQP4, AQP8 (Soria et al., 2009; Chen et al., 2014; Zhu, 2015). VIP can regulate the expression of AQP3, AQP4, AQP8 through the cAMP-PKA signaling pathway (Soria et al., 2009; Chen et al., 2014; Tan et al., 2021), thereby regulating the permeability of cell membranes to water. Our results also confirmed this view by showing the decreased VIP, 5-HT, cAMP, PKA, AQP3, AQP4 and AQP8 in the spring group.

In addition, 5-HT, VIP, cAMP, PKA have seasonal variation (Pfister and Storey, 2006; Mishra et al., 2018; Ciani et al., 2019; Li et al., 2020). For example, the concentration of 5-HT in the hippocampus of rats increases in summer and decreases in winter (Li et al., 2020). Therefore, the seasonal difference in intestinal water metabolism may be caused by seasonal changes of 5-HT and VIP, which regulate cAMP/PKA and affect the expression of AQPs. Our results also confirmed that the increased intestinal water volume in rats in summer may be caused by the increased 5-HT and VIP, and the upregulation of AQP3, AQP4 and AQP8 after cAMP/PKA activation. The decreased intestinal water volume in rats in spring may be caused by the decreased 5-HT and VIP, which inhibited cAMP/PKA, and then downregulated the expression of AQP3, AQP4 and AQP8. Combined with the results of this experiment, we believe that seasonal changes in intestinal water metabolism may be closely related to the changes in the regulation of AQPs by 5-HT and VIP through the cAMP-PKA signaling pathway.

On the other hand, the change in the water–liquid environment can affect microflora composition and abundance. Related studies have reported that the content and availability of water in the environment not only directly determines the lifespan of microorganisms (Or et al., 2007; Young et al., 2008) but also affects the nutrient diffusion and distribution range of microorganisms (Dechesne et al., 2008, 2010; Vos et al., 2013). For example, compared with the wet soil, the microbial diversity and abundance of *Enterobacterales*, *Clostridiales*, *Lactobacillales*, and *Bacteroidales* decreased significantly when the soil was dry, suggesting that the change in the water–liquid environment affects microflora composition and abundance (Šťovíček et al., 2017). Similarly, other studies have confirmed that the changes in intestinal water metabolism are consistent with the changes in microflora. For example, in the rat model of diarrhea-predominant IBS, AQP8 expression decreased, whereas intestinal flora imbalance appeared; in this case, the abundance of *Lactobacillus* significantly decreased, whereas that of *Clostridiales_bacterium* increased (Zhao et al., 2022).

Through the detection of rat fecal microbiota, differences in the abundance and composition of rat intestinal microflora were observed in different seasonal environments. The change in the season is mainly reflected through changes in temperature and humidity. Temperature and humidity have a close impact on intestinal microbiota (Sun et al., 2017; Deng et al., 2020). Therefore, we here mainly controlled temperature and humidity to simulate the environment of the four seasons. At the phylum level, the intestinal microflora of rats is similar to that of other mammals (including humans), including Firmicutes and Bacteroidetes (Ley et al., 2008). Our study revealed that no significant difference was observed in the flora structure of rats in different seasons at the gate level, and the two dominant bacteria were Firmicutes and Bacteroides, which may be due to the normal physiological state of rats. Under physiological conditions, some seasonal differences were observed in α and β diversities of the rat intestinal flora, although no statistically significant difference was noted. However, according to the PCoA results, the flora structure of the spring and autumn groups was similar. The proportion of Firmicutes and Bacteroidetes in the spring and autumn groups was similar. This may be due to similar temperature and humidity in spring and autumn. However, at the genus level, the dominant flora in different seasons exhibited significant differences, and numerous studies have confirmed this finding. For example, in a 2-year study of intestinal microflora of wild mice, a strong seasonal change was observed in the structure of mouse intestinal microflora. The abundance of *Lactobacillus* increased significantly in late spring/early summer and decreased significantly in late summer/early autumn. By contrast, the abundance of *Helicobacter* and *Alistipes* showed seasonal characteristics opposite to those of *Lactobacillus*, which increased significantly in late summer/early autumn and decreased significantly in late spring/early summer. However, the study attributed seasonal differences in bacteria to seasonal differences in the diet type (Maurice et al., 2015). In this study, we used laboratory rats as research objects, and diet types in different seasons were the same. However, our analysis of the genera level of intestinal flora in each group revealed that significant seasonal differences were still observed. For example, the abundance of *Lactobacillus* increased significantly in spring, while that of *Romboutsia* increased significantly in winter, indicating that the effect of season on intestinal flora is related to not only diet but also other factors. For example, some studies have shown that the amount of water and food intake of adult African giant rats is significantly higher in cold and dry seasons than in hot seasons (Dzenda et al., 2013), suggesting that different seasonal conditions affect water and food intake in animals (Ebling and Barrett, 2008; Ebling, 2015; Ahlberg et al., 2018). The difference in drinking water or food intake directly affects the digestion and metabolism of the host, including water and fluid metabolism in the intestinal tract. Therefore, this study directly focused on the difference in intestinal water metabolism with change in seasons, and analyzed the relationship between intestinal water metabolism and intestinal flora.

To better study the relationship between intestinal water metabolism and intestinal flora, on the basis of some related indicators of intestinal water metabolism, we analyzed the correlation between their content and the abundance of the top 25 bacteria at the genus level. To further determine the relationship between the seasonal difference in intestinal water metabolism and that in intestinal flora, we analyzed the correlation between the content of relevant indices of intestinal water metabolism and the dominant flora in different seasons. According to the results, the abundance of *Lactobacillus, Blautia, Faecalibaculum, Methanobrevibacter, Turicibacter, Alistipes,* and other bacteria in spring, autumn, and winter was negatively correlated with intestinal water content. The abundance of *Romboutsia*, *Helicobacter,* and other bacteria in summer was positively correlated with intestinal water content.

The therapeutic effect of *Lactobacillus,* a probiotic, in improving diarrhea has been widely reported (Li et al., 2021). The intestinal water content is higher and the abundance of *Lactobacillus* is significantly lower in IBS patients with diarrhea than in the healthy control group (Wang et al., 2020). *Blautia*, an anaerobic bacterium, widely exists in the feces and intestines of mammals, and 37°C is most suitable for its survival (Liu et al., 2021). The relative abundance of *Blautia* significantly increased with a decrease in intestinal moisture in patients with constipation (Botelho et al., 2020). By contrast, in patients with irritable bowel disease, the abundance of *Blautia* significantly decreased with the occurrence of mucous pus and bloody stool (Liu et al., 2021). Being a common bacterium in the gastrointestinal tract, *Faecalibaculum* is closely related to the occurrence of intestinal metabolic diseases. The relative abundance of *Faecalibaculum* was significantly decreased in diarrhea-predominant IBS patients, but significantly increased in mice with constipation (Liu et al., 2020; Chai et al., 2021). The abundance of *Methanobrevibacter,* a beneficial bacterium, was decreased in diarrhea pigs. After capsulized fecal microbiota transplantation, the diarrhea symptoms of piglets improved, and the abundance of *Methanobrevibacter* increased with a decrease in intestinal water content (Tang et al., 2020). *Turicibacter* is a gram-positive, anaerobic, non-spore-forming bacterium (Wang X. et al., 2021). After weaning calves recovered from bovine coronavirus-mediated diarrhea, the abundance of *Turicibacter* increased significantly (Kwon et al., 2021). *Alistipes* is a relatively new bacterial genus, typically isolated from the human gut microbiome (Parker et al., 2020). It requires high survival conditions when cultured *in vitro*, and therefore, its survival in liquid medium is difficult. Some studies have shown that the abundance of intestinal *Alistipes* in patients with Sjogren’s syndrome was significantly increased compared with that in patients with xerophthalmia (Moon et al., 2020). However, *Alistipes* was not observed in the intestine of diarrhea piglets (Huang et al., 2019), which may be because the dry environment of the intestine is more suitable for *Alistipes* survival. This, from the pathological perspecpective, confirms our results that the abundance of these bacteria is negatively correlated with intestinal water content. *Romboutsia* is a type of idiopathic anaerobe that is usually detected in the gastrointestinal tract of many vertebrates, including poultry (Maki et al., 2020). Studies have shown that after functional constipation is relieved with chrysanthemum polysaccharide, the relative abundance of *Romboutsia* increases with the recovery of the intestinal fluid (Wang J. et al., 2021). Similarly, *Helicobacter* is considered a pathogen and is associated with chronic diarrhea (Queiroz et al., 2013). This also confirms our findings from a pathological point of view that the abundance of these bacteria is positively correlated with the water content in the intestinal tract.

Therefore, we speculate that seasonal factors may cause seasonal changes in intestinal flora by affecting the intestinal water metabolism level in rats. *Romboutsia* and *Helicobacter* were positively correlated with intestinal water content. High temperature and humidity in summer enhanced the intestinal water metabolism level and intestinal water volume in rats, which may have led to an increase in the abundance of *Romboutsia* and *Helicobacter* as intestinal dominant flora in summer. However, *Lactobacillus*, *Faecalibaculum*, *Turicibacter,* and other bacteria were negatively correlated with intestinal water content. The temperature and humidity were relatively reduced in spring, autumn and winter, the intestinal water metabolism level and the intestinal water volume in rats decreased. At the same time, the abundance of *Lactobacillus*, *Faecalibaculum*, *Turicibacter,* and other bacteria increased, which also confirmed our speculation.

Notably, our present study only explores the difference in intestinal water metabolism caused by changes in environmental factors, such as temperature and humidity, with changes in in seasons. A certain correlation exists between this difference and the seasonal difference in intestinal flora. However, this correlation is not a simple linear relationship. For example, the abundance of *Romboutsia* exhibited a positive correlation with the AQP3 content, and had a positive correlation trend with fecal water content, but the abundance of *Romboutsia* increased in the winter environment relative to other seasons. In fact, the intestinal water metabolism level was weaker in winter than in summer. The factors and specific mechanisms affecting colonic water metabolism need to be further explored. At present, combined with studies in the same field, it has been found that mitophagy and oxidative stress participate in the seasonal physiological regulation of the body (Wang Y. et al., 2021). Stress or environmental changes can affect mitochondrial energy metabolism (Chang et al., 2023), and microbiota metabolites can also directly affect mitochondrial oxidative stress and the formation of mitochondrial autophagic lysosomes (Wang J. et al., 2022). Therefore, in the future, we will further explore the relationship between mitochondrial energy metabolism of intestinal cells and intestinal water metabolism, and explore the deep mechanism of adaptive changes in intestinal flora.

## Data availability statement

The data presented in the study are deposited in the NCBI-SRA repository (https://www.ncbi.nlm.nih.gov/bioproject/PRJNA908739), accession number PRJNA908739.

## Ethics statement

The animal study was reviewed and approved by Beijing University of Chinese Medicine Animal Ethics Committee (Approval number: BUCM-4-2,021,032,603-1,059).

## Author contributions

LL, TW, and SM performed the experiments. LL, JL, YS, and RW wrote the manuscript. TW, LS, KW, and HZ revised the manuscript. LL designed and supervised the study. All authors contributed to the article and approved the submitted version.

## Funding

This work was supported a grant provided by National Natural Science Foundation of China (Grant number: 8210152377).

## Conflict of interest

The authors declare that the research was conducted in the absence of any commercial or financial relationships that could be construed as a potential conflict of interest.

## Publisher’s note

All claims expressed in this article are solely those of the authors and do not necessarily represent those of their affiliated organizations, or those of the publisher, the editors and the reviewers. Any product that may be evaluated in this article, or claim that may be made by its manufacturer, is not guaranteed or endorsed by the publisher.

## Footnotes

^1^ https://www.tutiempo.net
